# The Effect of Physical and Chemical Cues on Hepatocellular Function and Morphology

**DOI:** 10.3390/ijms15034299

**Published:** 2014-03-11

**Authors:** Shimaa A. Abdellatef, Akihiko Ohi, Toshihide Nabatame, Akiyoshi Taniguchi

**Affiliations:** 1 Cell-Materials Interaction Group, Biomaterials Unit, Nano-Life Field, International Center for Materials Nanoarchitectonics (MANA), National Institute for Materials Science, 1-1 Namiki, Tsukuba, Ibaraki 305-0044, Japan; E-Mail: abdelaleem.shimaa@nims.go.jp; 2 Graduate School of Advanced Science and Engineering, Waseda University, 3-4-1 Okubo, Shinjuku-ku, Tokyo 169-8555, Japan; 3 MANA Foundry, International Center for Materials Nanoarchitectonics (MANA), National Institute for Materials Science, 1-1 Namiki, Tsukuba, Ibaraki 305-0044, Japan; E-Mails: ohi.akihiko@nims.go.jp (A.O.); nabatame.toshihide@nims.go.jp (T.N.)

**Keywords:** hepatocytes, topography, RGD, TiO_2_

## Abstract

Physical topographical features and/or chemical stimuli to the extracellular matrix (ECM) provide essential cues that manipulate cell functions. From the physical point of view, contoured nanostructures are very important for cell behavior in general, and for cellular functions. From the chemical point of view, ECM proteins containing an RGD sequence are known to alter cell functions. In this study, the influence of integrated physical and chemical cues on a liver cell line (HepG2) was investigated. To mimic the physical cues provided by the ECM, amorphous TiO_2_ nanogratings with specific dimensional and geometrical characteristics (nanogratings 90 nm wide and 150 nm apart) were fabricated. To mimic the chemical cues provided by the ECM, the TiO_2_ inorganic film was modified by immobilization of the RGD motif. The hepatic cell line morphological and functional changes induced by simultaneously combining these diversified cues were investigated, including cellular alignment and the expression of different functional proteins. The combination of nanopatterns and surface modification with RGD induced cellular alignment and expression of functional proteins, indicating that physical and chemical cues are important factors for optimizing hepatocyte function.

## 1. Introduction

Understanding the relationship between human cells and their natural environment is very important for tissue engineering. This is particularly true for liver cells, as they need a specific architecture with distinctive surroundings for optimum cellular function. A decrease in hepatocellular functionality generally occurs when liver cell lines are cultured on two-dimensional surfaces. Living cells in their native environment are embedded in a complex of well-defined organic material and macromolecules called the extracellular matrix (ECM). The ECM assembles into a three-dimensional mesh with precisely contoured nanostructures [1]. The architecture of the ECM provides essential physical cues and chemical factors that trigger optimum cellular behavior [2]. Collagen is an important protein of ECM that forms a triple helix structure to generate fibers approximately 300 nm long and 1.5 nm wide [3]. These helices are assembled into nanofibrillar networks that hierarchically extend for tens of micrometers in length and have diameters between 260 and 410 nm [4]. In addition to surface topography, the chemical composition of the ECM plays an essential role in the control of cellular behavior [5–7] through integrin-mediated signaling pathways [8,9]. Integrins are heterodimeric transmembrane surface receptors that mediate connections between cells and the ECM. Several ECM proteins activate integrins through different mechanisms, resulting in the integration of multiple signaling pathways and thus different cellular behaviors [10,11]. Fibronectin, for example, is an ECM protein with a specific peptide sequence, Arginine-Glycine-Aspartic acid (RGD), that alters cellular attachment and spreading, as well as cell integration properties [12,13]. Clearly, both topographical features and chemical stimuli afforded by the ECM provide essential cues that fundamentally manipulate cell morphology [14], leading to alterations in migration [15], proliferation [16], and cytoskeleton organization [17].

It is therefore critical to mimic these physical and chemical cues in order to maintain the required *in vivo* architecture and optimum cellular function [18–22]. An understanding of ECM simulation techniques, which collectively [23] or separately [24] use various types of cues, has been established at the cellular level. These techniques involve the use of co-culture systems [25], polymers [26], recombinant proteins [27] and inorganic compounds [28] and have resulted in the development of functionalized biomaterials that can optimize cellular behavior. Of particular interest is the use of TiO_2_ deposited thin films, which provide a significant interface for the regulation of hepatocyte cell line attachment and subsequent function [29]. Flexible surface modeling and biofunctionalization, as well as the ability to control thickness, are the main attractions of using these films in tissue engineering research. Consequently, the ability to manipulate a biomaterial to provide a controlled surface texture and chemical composition would allow cellular functions to be controlled, as this biomaterial would provide physical and chemical cues to trigger various biological responses.

The role of dimensional and geometrical characteristics of nanofeatures on hepatocellular behavior has been described in our recent research [30], in which topography manipulation showed an interesting role that involved the alteration of hepatic functionalities. This urged us to examine integration of another kind of ECM cues (*i.e.*, chemical cues) with the topography (physical cues), since physical and chemical cues have been proven independently to enhance hepatocellular behaviours. Consequently, such integration between physical and chemical cues will closely mimic the naturally occurring ECM cues. In detail, our aim was to study the collective influence of surface composition and topographical features of TiO_2_ on the biofunctionality of a hepatic cell line. Little attention has been focused on this to date, even though it is of clear importance for clarifying the relationship between unique ECM cues and optimum *in vitro* performance of liver cells. Amorphous TiO_2_ nanogratings with specific dimensional and geometrical characteristics (nanogratings 90 nm wide and 150 nm apart) were fabricated using electron beam lithography and atomic layer deposition. We believe that HepG2 cells recognize these specific TiO_2_ patterns (total dimension 240 nm). As a result of such recognition, the effective conditions for the optimization of the cellular behavior were maintained [30]. These TiO_2_ pattern highly mimic the variable diameters of the fibrillar structure of collagen, in which, the individual molecules of triple helical collagen are arranged into fibrils with diameter up to 10 nm, followed by a subsequent arrangement into greater fibrils with a larger diameter (several hundred nm) [31]. Furthermore, the incorporation of organic moieties with specified cellular functionality, such as RGD motifs, into the deposited TiO_2_ inorganic film was accomplished using an enzyme catalyzed oxidation reaction. The morphological and functional changes induced in hepatic cell lines by simultaneously combining the diversified cues were investigated; for example, cellular alignment and the production of different functional proteins were studied using fluorescent immunostaining techniques. An in-depth understanding of the relationship between various cues and hepatocyte behavior would lay the groundwork for this approach to be used in tissue engineering and bioreactor technology.

## 2. Results and Discussion

We examined the integration of chemical and physical cues to study the influence of such integration on the enhancement of hepatic cellular behavior. These assembled constructs mimic the native environment around cells. We used HepG2 cells, as they exhibit a lower cellular functionality upon culturing under flat tissue culture conditions [32] moreover, HepG2 is a stable cell line compared to primary hepatocytes, consequently the drawbacks associated with the alteration in the primary hepatocytes’ native culture environments upon their culture into a rigid flat substrate is avoided. Such alterations would result in a subsequent change in cell morphology, polarity and gene expression [33], which may cause discrepancies in results. Electron beam lithography (EBL) and atomic layered deposition (ALD) were used to produce dimensionally well-defined TiO_2_ nanogratings, and then the RGD peptide was immobilized. The effects of simultaneously combining these physical and chemical cues and their influence on HepG2 behavior were investigated over a short time course.

Alterations in the physicochemical characteristics of a biomaterial impact the modulation of cellular behavior [18–22] since surface chemistry [12] and substratum topography [34,35] each have profound effects. Recently, we examined the effect of manipulating physical cues on hepatic cellular behavior and showed that alterations in the shape or dimensional characteristics of physical cues critically affect optimum cellular function [30]. These alterations mimicked the physical cues of naturally occurring ECM components to control and regulate multiple hepatic functions. In the current study, we investigated the combined impact of diverse cues on hepatocellular behavior. The RGD peptide was immobilized on a TiO_2_ nanopattern to alter the hierarchically extended collagen nanofibrillar structure and hepatocellular behaviours were investigated. At first, cell viability upon culturing on TiO_2_ pattern and flat substrates was examined. The viability investigation was done using live/dead staining for intracellular esterase activity and cell membrane integrity. The use of pattern substrates showed no change in the viability (~98%) compared to flat surface (data not shown).

**Figure 1.**
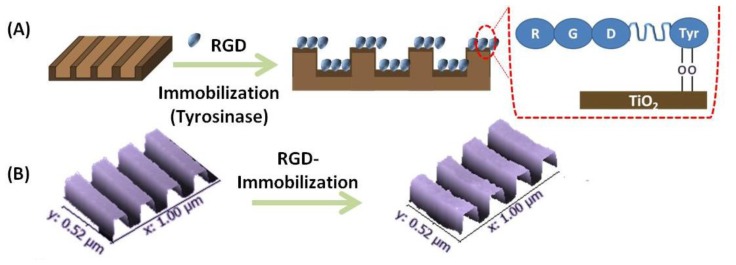

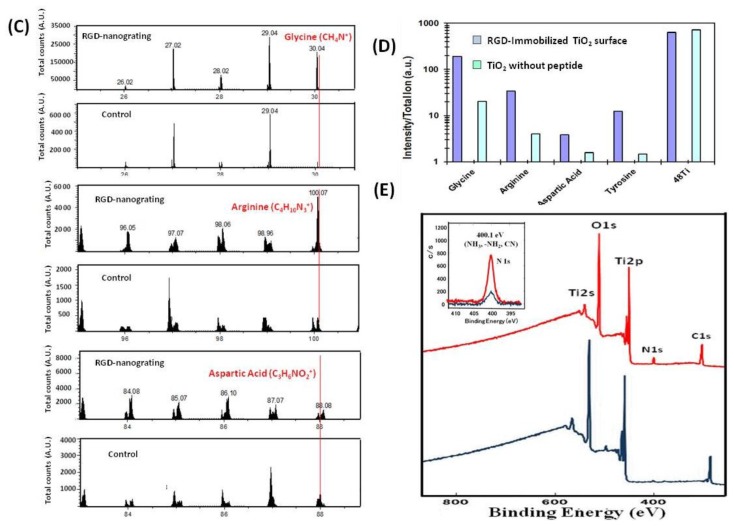
(**A**) Schematic showing the attachment of RGD on TiO_2_ nanogratings; (**B**) 3-D AFM images showing retention of the TiO_2_ nanopatterns after RGD immobilization; (**C**) Time-of-Flight Secondary Ion Mass Spectrometry (ToF-SIMS) confirmed the attachment of RDG peptide on the TiO_2_ surface. The specific fragment pattern generated by removal of individual amino acids from peptide is shown in the lower panels. (Glycine *m*/*z* 30.04 u:CH_4_N^+^, Arginine *m*/*z* 100.07 u:C_4_H_10_N^+^, Aspartic acid *m*/*z* 88.08 u:C_3_H_6_NO_2_^+^); (**D**) Typical peak ratio of amino acids normalized by total ions; (**E**) XPS spectra; wide scan of N1s spectra of TiO_2_ surfaces before and after the immobilization of peptide. Inset: a high-resolution scan of N1s.

The TiO_2_ nanogratings 90 nm wide and 150 nm apart were fabricated by EBL and ATD. The shape, dimensions and topographical features of the fabricated TiO_2_ nanopatterns were characterized before and after immobilization of the peptide by atomic force microscope (AFM) (Figure 1B). The shape and dimensions of the nanogratings were not affected by peptide immobilization, which is important, as the maintenance of topography was a key concern in this study. Peptide immobilization and surface characteristics were confirmed using time of flight-secondary ion mass spectroscopy (TOF-SIMS) and X-ray photoelectron spectroscopy (XPS) (Figure 1C–E). Typical amino acids produced by fragmentation of the peptide (*i.e.*, Glycine, Arginine, Aspartic acid) were assigned by their mass spectral signals and are presented in Figure 1C. These characteristic fragments were normalized and compared to the control (The TiO_2_ substrate that was subjected to the same procedure without presence of peptide) to determine coupling efficiency (Figure 1D). The chemical atomic composition of the RGD-immobilized TiO_2_ substrate was investigated using XPS. Figure 1E shows the appearance of N1s signals after peptide immobilization to TiO_2_ surfaces, which are absent in the control. High-resolution N1s spectra that confirmed the immobilization of RGD on the TiO_2_ substrate is shown in the inset in Figure 1E. Thus, maintenance of fidelity and uniformity of the TiO_2_ nanopattern after RGD immobilization was confirmed.

### 2.1. Influence on Functional Protein Expression

The expression of hepatic functional proteins is stimulated by naturally occurring ECM. Therefore, we examined the integration of physical and chemical factors simulating native ECM to optimize cellular behavior. The influence of topography and surface chemistry on cells was determined by investigating TiO_2_ substrates that combine both factors. We observed changes in the production level of functional proteins such as albumin, transferrin, and cytochrome P-450 using fluorescence immunostaining techniques. Since the pattern and flat areas exist in the same substrate, it is difficult to separate cells; further quantification such as RT-PCR or ELISA, were therefore not performed.

#### 2.1.1. Albumin

**Figure 2.**
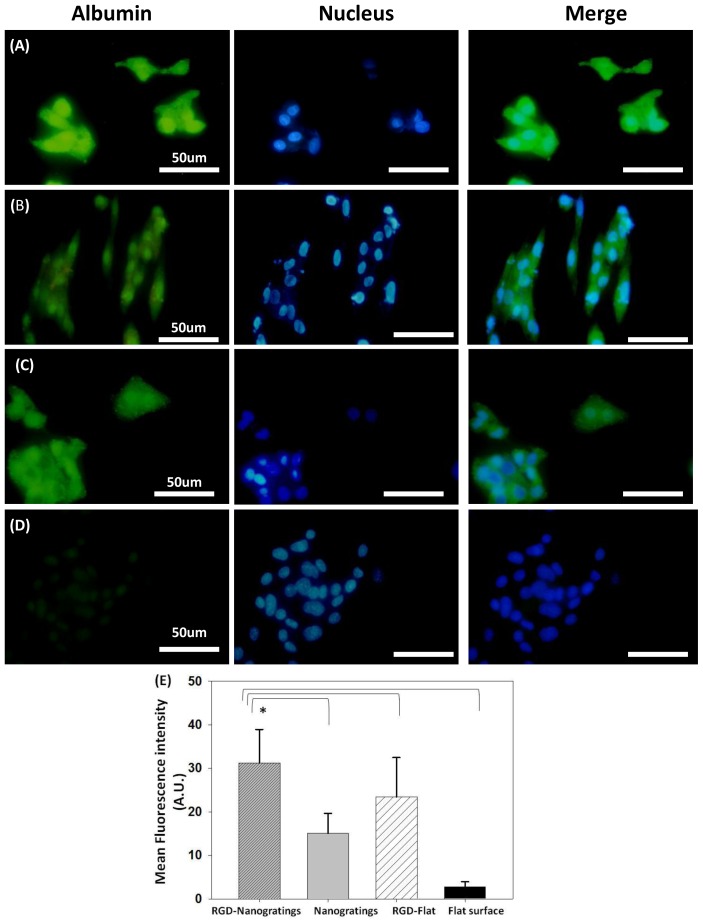
Immunofluorescence staining of the hepatic functional protein, albumin (green excitation λ = 448 nm), in HepG2 cells cultured on (**A**) RGD TiO_2_-nanograting pattern; and (**B**) TiO_2_-nanograting pattern alone; and (**C**) RGD-flat surface compared to cells cultured on (**D**) Flat surface alone (control); (**E**) Relative mean fluorescence intensity calculated using Image J for HepG2 cultured on biofunctionalized TiO_2_ substrates * Statistical significance at (*p* < 0.05).

Fluorescence immunostaining of albumin was performed after HepG2 seeding for 12 h, followed by examination of green fluorescent albumin under a fluorescence microscope. Figures 2 and S1 show fluorescence micrographs of HepG2 cells seeded for 12 h on various TiO_2_ substrates with and without RGD immobilization. Changes in the chemical composition of the substrate by immobilization of the RGD peptide increased the production of cytoplasm and nucleus [36] localized albumin compared to substrate without RGD. Furthermore, modulation of TiO_2_ topography while maintaining the same surface chemical functionality considerably increased the production of albumin (green fluorescence) in HepG2 cells. A quantitative comparison of albumin expression is presented in Figure 2E, in which the calculated fluorescence intensities obtained from HepG2 cells cultured on RGD-TiO_2_ nanogratings were statistically compared to the fluorescence intensity obtained from TiO_2_ nanopatterned surface alone, an RGD-flat surface, or a flat surface alone. RGD functionalized nanogratings showed a significant increase in albumin expression (*p* < 0.05). Thus, the results suggest that the 240 nm gratings on the TiO_2_ substrate topography functionalized with RGD closely mimic the natural environment essential for the optimum production of albumin by HepG2 cells.

#### 2.1.2. Transferrin

**Figure 3.**
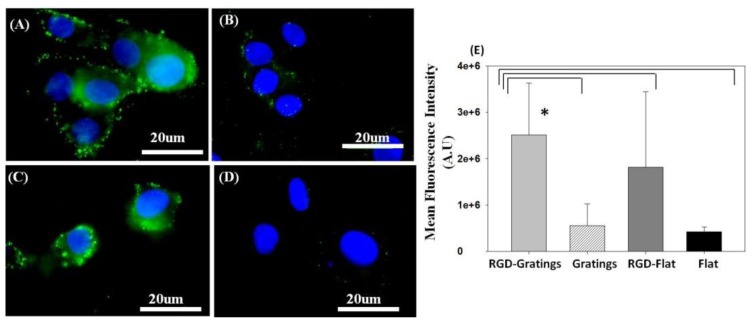
Immunofluorescence staining of the hepatic protein, transferrin (green excitation λ = 448 nm), and nucleus (blue) in HepG2 cells cultured on (**A**) RGD TiO_2_-nanograting pattern; and (**B**) TiO_2_-nanograting pattern compared to cells cultured on (**C**) RGD-flat surface and (**D**) Flat alone (control); (**E**) Relative mean fluorescence intensity calculated using ImageJ for HepG2 cultured on various biofunctionalized TiO_2_ substrates. * Statistical significance at (*p* < 0.05).

The production of the functional protein transferrin was determined as green fluorescence using an immunostaining technique. Figures 3 and S2 show fluorescence images of transferrin synthesized upon culturing HepG_2_ on various TiO_2_ substrates. Transferrin production significantly increased (*p* < 0.05) 12 h after seeding HepG2 on the RGD-nanogratings (Figure 3A), while the TiO_2_ nanopattern without RGD showed lower transferrin levels (Figure 3B). Furthermore, the biofunctionalized flat surface showed a reduction in the production of transferrin compared to the biofunctionalized pattern substrate (Figure 3C). Although transferrin expression is expected in HepG2 cells, the flat surface alone without RGD showed minimal transferrin production when compared relatively to the RGD-substrate (Figure 3D). A quantitative comparison of transferrin expression is presented in Figure 3E, in which the calculated fluorescence intensities obtained from HepG2 cells cultured on RGD-TiO_2_ nanogratings were statistically compared to the fluorescence intensity obtained from TiO_2_ nanopatterned surface alone, an RGD-flat surface, or a flat surface alone. RGD functionalized nanogratings showed a significant increase in transferrin expression (*p* < 0.05), thus, emphasizing the role of RGD as a chemical nanocue in addition to the physical topography cues. These results suggest that the level of transferrin is modulated through mimicking the natural ECM cues, such as topography and chemical factors.

#### 2.1.3. Cytochrome P-450

**Figure 4.**
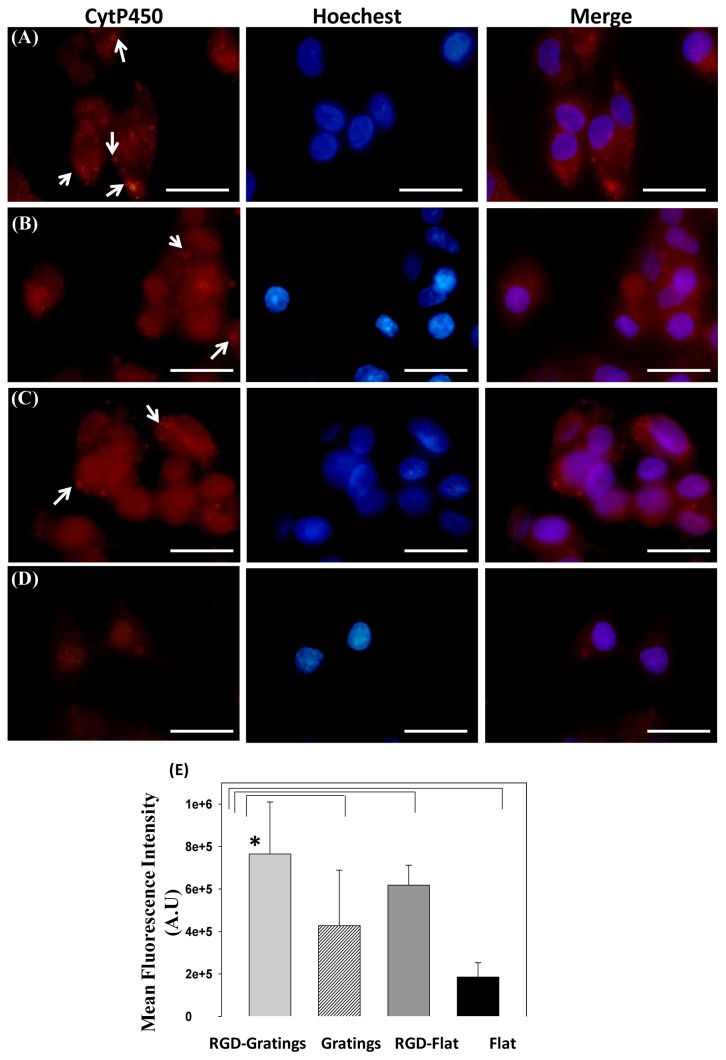
Immunofluorescence staining of the hepatic protein, cytochrome P-450 2C9 (red excitation λ = 578 nm), in HepG2 cells cultured on (**A**) RGD TiO_2_-nanograting pattern; and (**B**) TiO_2_-nanograting pattern; (**C**) RGD-flat surface compared to cells cultured on (**C**) and (**D**) control, arrow heads indicate the fluorescent red dots of cytochrome P-450; (**E**) Relative mean fluorescence intensity calculated using ImageJ for HepG2 cultured on biofunctionalized TiO_2_ substrates. * Statistical significance at (*p* < 0.05).

Cytochrome P-450 proteins are a family of hemoproteins found prominently in liver, whose members catalyze the metabolism of a variety of endogenous and xenobiotic substrates [37]. Specifically, cytochrome P-450 2C6 is expressed naturally in the rat liver cells without the presence of specific chemical inducer (*i.e.*, phenobarbital) [38] while its counterpart cytochrome P-450 2C9 is expressed in human liver cells. Thus, the production of cytochrome P-450 2C9 was analyzed after culturing HepG2 cells on RGD-TiO_2_ nanopatterned and flat substrates. Figures 4 and S3 show the formed cytochrome P-450 as red fluorescence inside HepG2 cells cultured on TiO_2_ substrates after 18 h. Cells cultured on RGD-TiO_2_ nanogratings showed a significant increase in cytochrome P-450 (Figure 4A) compared to cells seeded on the nanopatterned, RGD-flat surface, or flat surface (Figure 4B–D). A quantitative comparison of cytochrome P-450 expression is presented in Figure 4E, in which the calculated fluorescence intensities obtained from HepG2 cells cultured on RGD-TiO_2_ nanogratings were statistically significant compared to the fluorescence intensity obtained from TiO_2_ nanopatterned surface alone, an RGD-flat surface, or a flat surface alone. RGD functionalized nanogratings showed a significant increase in cytochrome P-450 expression (*p* < 0.05). These results suggest that the manipulation of topography can induce a change in the production of cytochrome P-450 levels while preserving the same surface biocharacteristics. Furthermore, it indicated the role of chemical cues (RGD) in the stimulation of hepatocellular functionalities.

An increase in the expression of the important functional proteins albumin, transferrin, and cytochrome P-450 was determined. A significant enhancement in expression of these liver-specific markers on RGD coated surfaces was observed compared to uncoated TiO_2_ substrates, regardless of the topography of the substrate. This could be due to the activation of an integrin-dependent intracellular signaling pathway. Alterations in the surface chemical composition of biomaterials modulate charge, hydrophilicity, and protein adsorption, with subsequent alteration in cellular affinity for the substrate [39,40]. The TiO_2_ substrate that integrated nanotopography with the adhesive peptide motif maximally increased most hepatocellular functionalities tested. The synergistic effect of combining both chemical and physical cues provides the optimum cell integrity and orientation, with a subsequent activation of specific cell membrane integrins. Thus, the better the ECM intrinsic elements are mimicked, the better the substrate’s cytocompatibility and the functionality of HepG2. Less of an effect was observed for transferrin expression, as hepatic cell functionality mainly depends on intricate signals and pathways that are activated by diversified cues. The detailed mechanisms underlying such synergism, and identification of their essential regulating factors, require further study. However, we can conclude that chemical cues play a major role in the enhancement of hepatic functionality compared to substratum topography alone, and the integration of both cues has a synergistic effect on most hepatic functionalities. Thereby, our findings suggest that surface nanofeatures with well-defined dimensions and chemical composition that imitate ECM fibrillar structures and containing a specific motif (e.g., RGD) could be utilized to increase multiple cell functions, rather than simply using nanotopography or chemical cues alone.

### 2.2. Influence on the Structure of Hepatic Cell Line (HepG2)

Alterations in TiO_2_ topography and chemical surface characteristics could greatly influence cellular orientation, alignment and elongation, in turn regulating cellular behavior. Thus, actin filament rearrangements and integrin mediated focal adhesions were studied.

#### 2.2.1. Cell Alignment and Actin Filament Rearrangement

**Figure 5.**
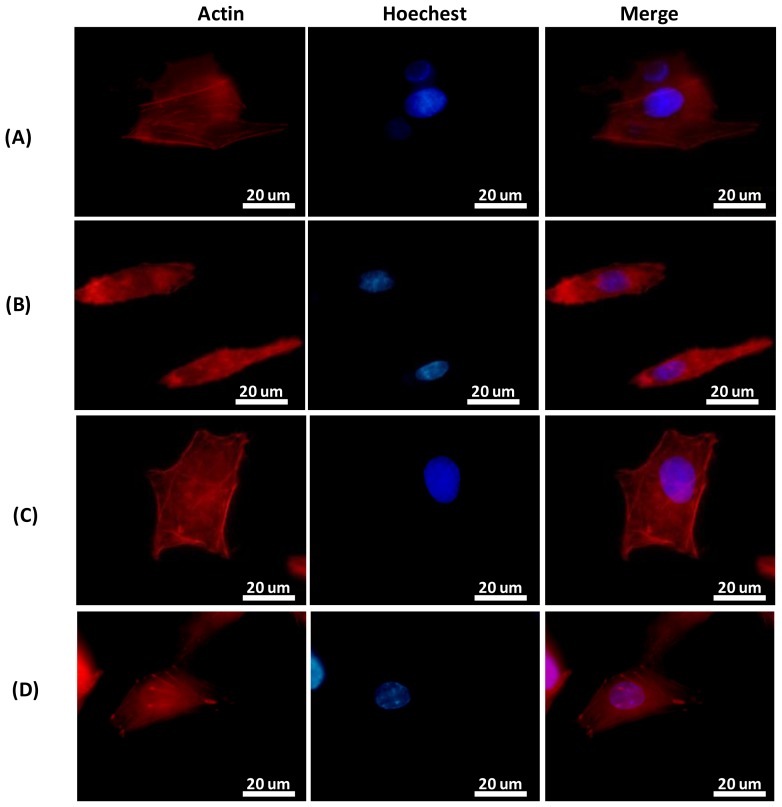
Actin fluorescent staining to study the cell alignment and cytoskeletal rearrangement of HepG2 cells cultured for 12 h on (**A**) RGD-TiO_2_ nanogratings compared to (**B**) TiO_2_ nanopattern alone, on which the actin filaments are arranged parallel to the TiO_2_ nanofeatures (**C**) RGD-TiO_2_ flat surface and (**D**) Flat surface alone or control.

Since cell spreading and alignment mainly rely on the surface characteristics of the biomaterial, the concurrent effect of immobilization of RGD on the TiO_2_ surface on the cytoskeleton, actin filament assembly and reorganization were observed. HepG2 cells cultured on dimensionally well-defined TiO_2_ nanograting substrates or flat surfaces with and without RGD biofunctionalization were examined using fluorescence staining and SEM. Figure 5 shows the fluorescence staining of actin filaments. The staining indicates that the structural reorganization of HepG2 cells to some extent parallels the TiO_2_ nanogratings, while less organized cytoskeletons are observed in cell cultures grown on RGD-flat surfaces. Moreover, the width of cells increased when cultured on an RGD-functionalized surface compared to the nanopatterned substrate alone or on a flat surface (Figure 5C,D). RGD-immobilized TiO_2_ substrates resulted in an increase in cell spreading (width), and a decrease in cell extension (length) compared to TiO_2_ alone, suggesting enhanced cell-substratum affinity. These alterations may be mediated by the presence of adhesive ligands that encourage the organization of three dimensional actin cytoskeletons; actin filament reorganization is mediated by the assembly of intracellular and extracellular complex domains (integrins) that bridge adhesive ligands (RGD) and the actin cytoskeleton.

**Figure 6.**
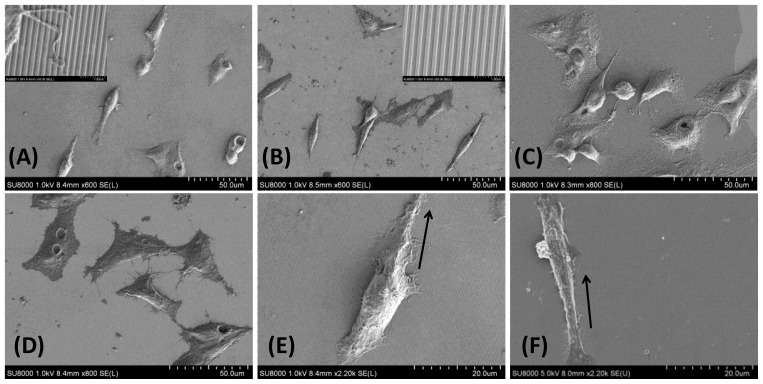
SEM images of HepG2 cells cultured for 12 hrs in (**A**) RGD-TiO2 nanogratings compared to (**B**) TiO_2_ nanopattern alone and (**C**) RGD-TiO_2_ flat surface (**D**) flat surface alone (Control), insets are higher magnification SEM images that show the direction of nanogratings and the cell alignments parallel to TiO_2_ lines. Furthermore, the increase in cell width where indicated in (**E**) RGD-TiO_2_ and (**F**) nanogratings alone while black arrow indicate the direction of nanogratings.

Further, cellular alignment was investigated using scanning electron microscope. Figure 6 shows cellular attachments parallel to the longitudinal axes of TiO_2_ gratings while such arrangements cannot be observed in the flat surface with or without RGD. Moreover, the biofunctionalization of the TiO_2_ nanopattern resulted in an increase in cellular spreading when cultured on an RGD-functionalized surface compared to the nanopatterned substrate alone (Figure 6E,F). The integration of both surface chemistry and nanotopography enhanced cell spreading compared to nanogratings alone, resulting in cells aligned along the long axis of the underlying topographical features. These observations suggest that the presence of chemical cues such as the RGD motif result in increased substrate affinity and play a dominant role in cellular morphology, as do physical nanocues such as topography. Thus, the presence of discrete surface cues is essential for selective cell adhesion and activation, indicating that these cues have clear applications in regenerative medicine.

#### 2.2.2. Integrin Mediated Focal Adhesion

**Figure 7.**
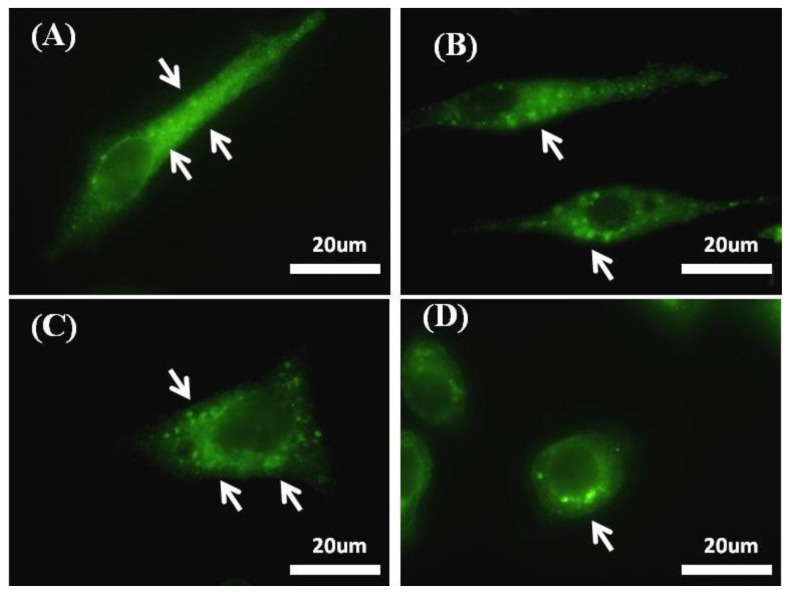
An increase in integrin β1 clusters that mediate focal adhesion formation using immunostaining of integrin B1 by Alexa-fluor 488 linked-AB (excitation λ = 488 nm) of HepG2 cells cultured on RGD-TiO_2_ substrates on which (**A**) RGD-nanogratings show high integrin β1 clusters while (**B**) Nanogratings alone show low integrin B1 clustering compared to it, on the other hand (**C**) RGD-flat surface shows high integrin β1 clusters, mean while (**D**) flat substrate alone (control) has the lowest integrin clustering; arrow heads show clusters of integrin B1 as green fluorescent dots.

Integrin β1 mediated focal adhesion was compared on the nanograting surface and the flat surface without or with RGD using immunofluorescence staining of a transmembrane protein, integrin β1. Integrin-mediated signals are leading mediators for controlling cellular behavior, adhesion, and functionality. Cellular behavior is dependent on integrin interactions with the substratum: the integrin extracellular domains bind to a specific motif on the ECM, and their intracellular domains are associated with the actin cytoskeleton and affiliated proteins. Biochemical motifs, as well as topographical characteristics, such as dimension, shape, symmetry, and roughness separately play dominant roles in integrin clustering and focal adhesion formation [41–43]. Figure 7 shows the expression of integrin β1 clusters as green fluorescence upon culturing HepG2 cells on TiO_2_ nanogratings with a total dimension of 240 nm and flat surface substrates with and without immobilization of RGD for 12 h.

An increase in integrin β1 was observed after seeding HepG2 cells on the RGD immobilized continuous grating compared to seeding on the nanopattern alone (Figure 7A,B) Thus, the integration of chemical and physical nanocues synergistically facilitates integrin clustering (Figure 7A) and further recruits proteins to focal adhesions. Although the synergistic effects are enhanced compared to each independent nanocue, individual topographical or chemical nanocues showed comparable stimulation of integrin clustering (Figure 7B,C). This emphasizes the role of surface characteristics and the substratum interface for the design and fabrication of biomimicry constructs with enhanced biofunctionality.

#### 2.2.3. Alteration in Natural Extracellular Matrix Assembly

**Figure 8.**
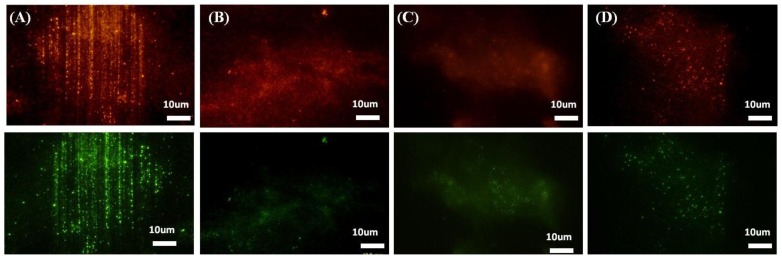
ECM components assembled by HepG2 cells cultured for 66 h on TiO_2_ substrates. (**A**) RGD-nanogratings; (**B**) nanogratings alone and compared to (**C**) RGD-flat surface and (**D**) flat substrate or control alone. Red fluorescence represents collagen IV Ab (2ry-Alexa fluor-540), green fluorescence represents fibronectin (2ry Ab Alexa-fluor-488) after decellularization using H_2_O for 1 h at 37 °C.

The influence of nanopatterns and RGD immobilization on two naturally formed and assembled ECM components, collagen IV and fibronectin, was studied (Figure 8). Decellularization was thus performed and changes in the assembly of fibronectin and collagen IV were examined using an immunostaining technique. Figure 8 shows the alteration in the assembly and alignment of native fibronectin and collagen IV after culturing and decellularization of HepG2 cells on an RGD-functionalized TiO_2_ nanopatterned surface relative to the nanopatterned substrate alone, RGD-flat surface, and flat surface alone. The assembly of fibronectin and collagen IV was considerably stimulated and aligned in the planar area between the nanogratings on the RGD-functionalized nanopatterned surface compared to other substrates (Figure 8A). RGD alone, acting as a chemical cue, enhanced cell-substratum affinity, resulting in the up-regulation of the expression of native assembled ECM components. The presence of nanogratings that resemble collagen fibrils acted as vertical ledges in between the planar areas. This topography increased the assembly of ECM components and aligned them in between these ledges. Although integration of RGD with topography showed aligned structures while other substrates showed random structures, the dimensions and size of assembled proteins cannot be recognized from such experiments. Consequently, the collective role of physical and chemical cues profoundly altered the active assembly of ECM components, resulting in further modification in tissue reorganization.

## 3. Experimental Section

### 3.1. Immobilization of RGD on TiO_2_ Nanopatterned Substrates

The fabrication of nanopatterned TiO_2_ substrate using electron beam lithography and atomic layer deposition was previously reported [30]. Cleaned Si(100) substrates were coated with ZEP520A resist (Nippon Zeon Co., Tokyo, Japan) and a thinner (anisole) at a ratio of 1:2 using a spin coater (Mikasa 1H-D7, Tokyo, Japan) at 6000 rpm. Prebaking was done at 180 °C for 3 min, followed by spin coating with a very thin layer of conductive material (10–20 nm) (Espacer; Syowa Denko Co., Tokyo, Japan) at 2000 rpm. The substrate was then irradiated with an e-beam (Elionix ELS-7500EX, Tokyo, Japan) with acceleration voltage of 50 kV and an I beam amperage of 220 pA. The precise size of the fabricated substrate resulting from each e-beam was confirmed using SEM. The substrate was then developed using H_2_O, *N*-amyl acetate and methyl isobutyl ketone (89%)/isopropyl alcohol (11%) (Wako Co., Tokyo, Japan) and dried with N_2_ gas. An etching step was performed using inductively coupled plasma-reactive ion etching at 50 W (sulphur hexafluoride 2.5 cc s^−1^ + methyl tetrafluoride 3.5 cc s^−1^) with a total pressure of 0.1 Pa for 101 s. Next, the resistance was removed using O_2_ plasma, DMAC (dimethyl acetamide) and SPM (H_2_SO_4_ + H_2_O_2_, 3:1), respectively. The next steps included coating with a photoresist (AZ-5214E), UV irradiation with a photomask, reversal baking at 120 °C, and flood exposure to UV. The substrate was then developed using HMD (hexamethyl disilazane) and 2.38% TMAH (tetramethyl ammonium hydroxide) (Wako Co., Japan) for 1 min, and then rinsed with H_2_O. Finally, atomic layer deposition was conducted (Picosun SUNALE R-150, Masala, Finland). The deposition pressure inside the chamber was 500 Pa at a temperature of 100 °C. The thickness of the TiO_2_ layer was controlled by the number of cycles: 70 cycles gave a thickness of 5 nm. The TiO_2_ precursor (tetra(dimethylamino)titanate) was pumped into the chamber, followed by argon gas to remove the undeposited precursor. Next, H_2_O vapour was pumped in to form the inorganic TiO_2_ layer from the organic precursor, and then argon gas was pumped in to remove residual H_2_O. The fabricated substrate was washed by sonication in an ethanol and acetone solution for 15 min and dry heat sterilized at 170 °C for 1 h. The RGD peptide (Proline-Arginine-Glycine-Aspartic acid-Glycine-Glycine-Glycine-Glycine-Glycine-Tyrosine) was purchased from Chinese Peptide Company (Hangzhou, China). The purity of the peptide was 95.9% as determined by high performance liquid chromatography (HPLC) and mass spectrometry. Tyrosinase from mushroom (lyophilized powder, ≥1000 units/mg solid; Sigma-Aldrich, St. Louis, MO, USA) was used for the oxidative reaction. All reagents were dissolved in PBS (diluted 1:10) and sterilized by filtration (0.2 μm Minisart CE, Satorius Stedim, Gottingen, Germany).

The immobilization of peptide on the TiO_2_ substrate used an enzyme catalyzed oxidative reaction to link the peptide to the functionalized surface, as reported previously [44]. First, sterilized TiO_2_ substrates were immersed in peptide solution (100 μg/mL) and tyrosinase solution (400 μ/mL) was added. The immobilization reaction was carried out for 15 min, and then the peptide-immobilized substrates were removed, washed by immersion in distilled water, and air-dried. TiO_2_ nanopatterned substrates before and after RGD immobilization were characterized by atomic force microscopy (SII NanoTechnology L-Trace, Tokyo, Japan) and time of flight-secondary ion mass spectroscopy using a TRIFT V (YLVAC-PHI, Chigasaki, Japan). Fragment patterns generated from amino acids allowed confirmation of the attached surface peptides. The RGD conjugated TiO_2_ substrates were characterized by X-ray photoelectron spectroscopy using a PHI Quantera SXM (ULVAC-PHI, Chigasaki, Japan).

Fabricated TiO_2_ nanopatterned surfaces were characterized using a scanning electron microscope (Hitachi-S3000N, Tokyo, Japan) and atomic force microscopy.

### 3.2. Hepatic Cell Line Culture

HepG2 were cultured in Dulbecco’s MEM (Nacalai Tesque, Kyoto, Japan) with 10% heat inactivated FBS and supplemented with 100 U penicillin/100 ug streptomycin (Nacalai Tesque, Kyoto, Japan) per ml medium. All cells were maintained at 37 °C in a 100% humidified atmosphere under 5% CO_2_. At 70%–80% confluency, cells were trypsinized and seeded over the TiO_2_ nanopatterned surfaces for 12 or 18 h. Low density cell seeding were used to enable the examination on a almost single cell-basis. For the expression of ECM components, cells were cultured for 66 h with the medium replaced every 33 h. Each experiment was repeated at least three times.

### 3.3. Immunostaining and Fluorescence Detection

HepG2 cells cultured on TiO_2_ nanopatterned substrates were fixed for 15 min in 4% P-formaldehyde (PFA) in PBS at 4 °C and excess aldehyde was quenched using 0.1 M glycine for 5 min. Cells were permeabilized with 1% triton X-100 in PBS for 5 min, and then primary antibodies were added: rabbit polyclonal anti-albumin antibody-*C*-terminal diluted 1:25 (Abcam, Cambridge, UK), rabbit polyclonal anti-transferrin antibody diluted 1:500 (Abcam, Cambridge, UK), mouse monoclonal anti-cytochrome P-450 2C6 antibody 1 ug/mL (Abcam, Cambridge, UK), CD29 mouse anti-human mAb-ALexa Fluro^®^ 488 conjugate diluted 1:100 (Life Technologies Invitrogen, Eugene, OR, USA) for integrin B1 at 4 °C overnight. Next, secondary antibodies were added for 1 h: Alexa Fluor^®^ 568 goat anti-mouse IgG (H + L) (Heavy + Light chains) diluted 1:500 (Life Technologies Invitrogen, Euogenes, OR, USA) or goat polyclonal anti-rabbit IgG-H&L-DyLight^®^ 488 diluted 1:500 (Abcam). Observation was performed using an upright fluorescence microscope (Olympus BX51, Tokyo, Japan) equipped with an Olympus DP70 digital camera. DP Controller Ver. 2.1.1, (Olympus corporation, Tokyo, Japan) was used to process the images. Quantitative comparison of albumin expression was determined using the freeware image analysis software, Image J, WS Rasband, National Health Institute, Bethesda, MA, USA) as previously reported [45]. Cell area was determined by manual delineation of raw fluorescence images. A minimum of 12 cells were analyzed from two independent experiments. Cytoskeletal F-actin was visualized by treating the cells with phalloidin-TRIC (Sigma-Aldrich, St. Louis, MO, USA) at 5 μg/mL for 15 min. Nuclei were stained with Hoechst 33342 at 5 μg/mL for 10 min.

To investigate ECM components, decellularization was performed as previously reported [46] on cells cultured on TiO_2_ nanopatterned surfaces for 66 h, followed by incubation with 2 mL of distilled water for 1 h at 37 °C. TiO_2_ nanopatterned surfaces were washed by immersion in PBS. The deposited ECM was fixed using 4% PFA for 20 min, followed by permeabilization with 0.5% triton X-100 in PFA. Primary antibodies such as mouse monoclonal antifibronectin diluted 1:50 (Santa Cruz Biotechnology Inc., Santa Cruz, CA, USA) and mouse monoclonal anticollagen IV diluted 1:50 (Santa Cruz Biotechnology Inc.) were added for 30 min, and then secondary antibodies (goat anti-mouse IgG-TR diluted 1:250 (Santa Cruz Biotechnology Inc.), Alexa Fluor^®^ 488 goat anti-mouse IgM (H+L) diluted 1:250 (Life Technologies Invitrogen, Euogenes, OR, USA) were added for 30 min. The effective removal of cells and cellular debris by this method was verified by fluorescence labelling of the decellularized TiO_2_ nanopatterned surfaces using Hoechst 33342.

### 3.4. Scanning Electron Microscope Investigation

Investigations of the cell rearrangements and alignments were done by scanning electron microscopy (SEM) using (Hitachi S-4800, Tokyo, Japan). Prior to SEM investigations the samples were prepared as follows. At the end of the culture period the cells on the TiO_2_ substrates were fixed with 2.5 vol % glutaraldehyde (Wako, Japan) in PBS for 2 h. After which the cells were dehydrated through a series of ethanol concentrations (10%, 40%, 60%, 80%, 100%) for 5 min, respectively. Final desiccation was done using freeze drying (Hitachi Es 2030, Tokyo, Japan). Finally, the samples were placed on SEM specimen holders and observed under field-emission scanning electron microscope operated at an acceleration voltage in the range 1–5 kV.

## 4. Conclusions

The collective role of chemical (RGD) and physical (topography) cues on the behavior of hepatic cells was studied. TiO_2_ substrates that simultaneously integrate nanotopography with an adhesive peptide motif (RGD) maximally increased hepatocellular functionality. A significant enhancement in the expression of liver-specific markers was observed on RGD-coated surfaces compared to uncoated substrates. These results emphasize the major role of chemical cues in enhancing hepatic functionality compared to substratum topography alone. The manipulation of chemical cues and/or distinct topographical features caused modifications in cellular reorganization mediated by changes in cellular attachments, spreading, integrin clustering and assembly of ECM components. Thus, we conclude that modifications in the physicochemical characteristics of biomaterials, either independently or by combining both chemical and physical cues, optimize cell integrity and orientation, with subsequent alterations in the behavior of hepatic cells. These findings could be utilized in the design of bio-inspired functionalized constructs for hepatic bioreactors for tissue engineering and other applications.

## Supplementary Information
