# Supplementary material for: The Effect of Physical and Chemical Cues on Hepatocellular Function and Morphology
Source: Int J Mol Sci. 2014 Mar 11;15(3):4299–317. doi: 10.3390/ijms15034299 (PMC3975399; doi:10.3390/ijms15034299)

Supplementary Information

Figure S1. Immunofluorescence staining of the hepatic functional protein, albumin (green excitation λ = 448 nm), in HepG2 cells cultured on (**A**) RGD TiO_2–nanograting pattern and (**B**) TiO_2–nanograting pattern alone and (**C**) RGD-flat surface compared to cells cultured on (**D**) Flat surface alone (control).

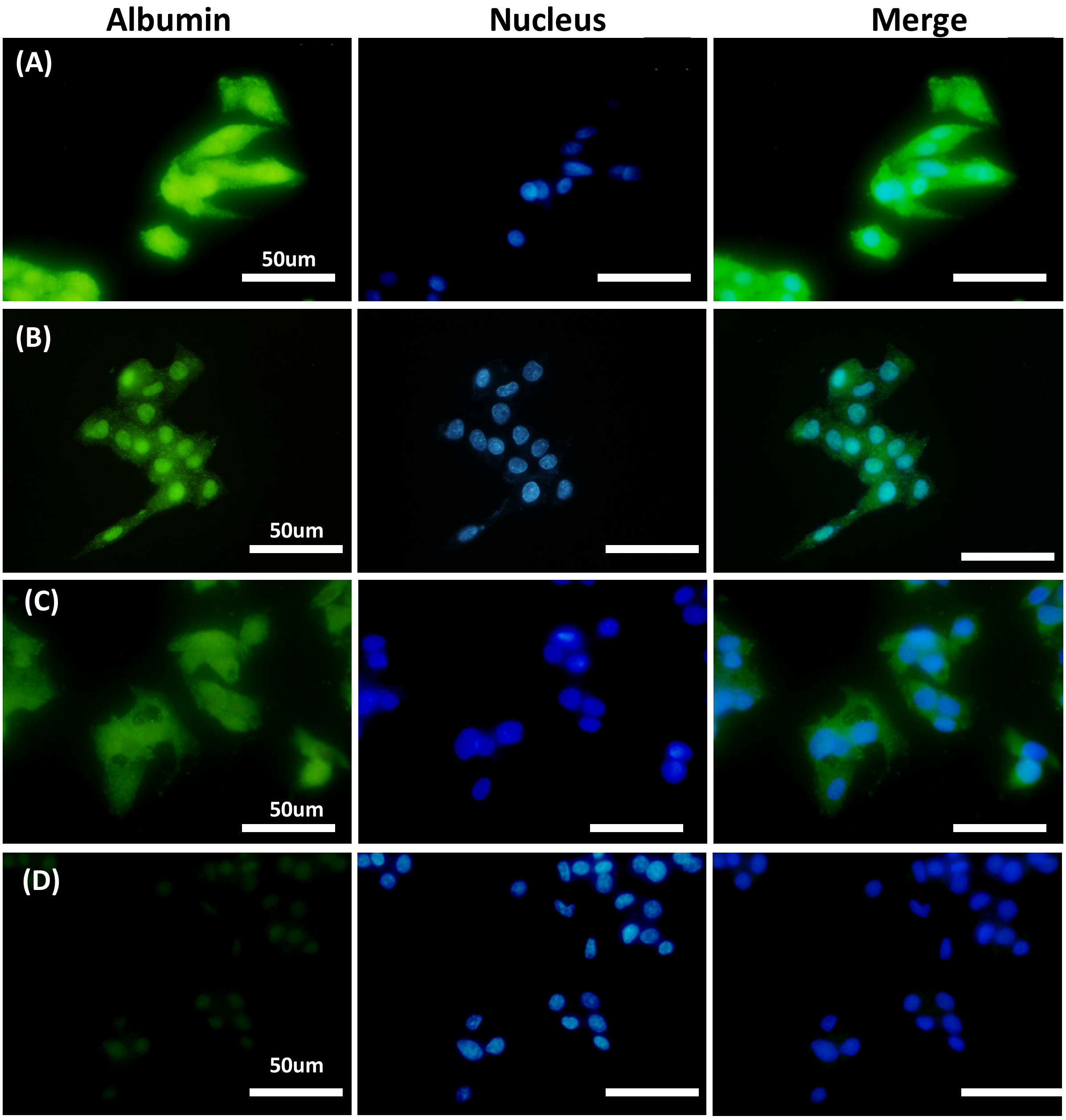

Figure S2. Immunofluorescence staining of the hepatic protein, transferrin (green excitation λ = 448 nm), and nucleus (blue) in HepG2 cells cultured on (**A**) RGD TiO_2–nanograting pattern and (**B**) TiO_2–nanograting pattern compared to cells cultured on (**C**) RGD-flat surface and (**D**) Flat alone (control).

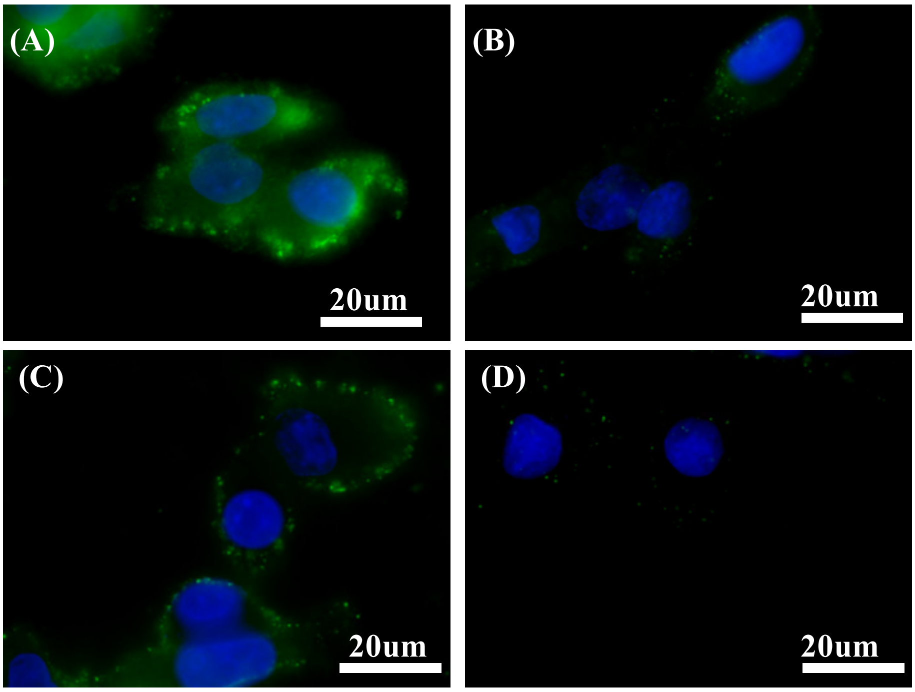

Figure S3. Immunofluorescence staining of the hepatic protein, cytochrome P450 (red excitation λ = 578 nm), in HepG2 cells cultured on (**A**) RGD TiO_2–nanograting pattern and (**B**) TiO_2–nanograting pattern; (**C**) RGD-flat surface compared to cells cultured on (**C**) and (**D**) control, arrow head indicates the red fluorescence of cytochrome P450 2C.

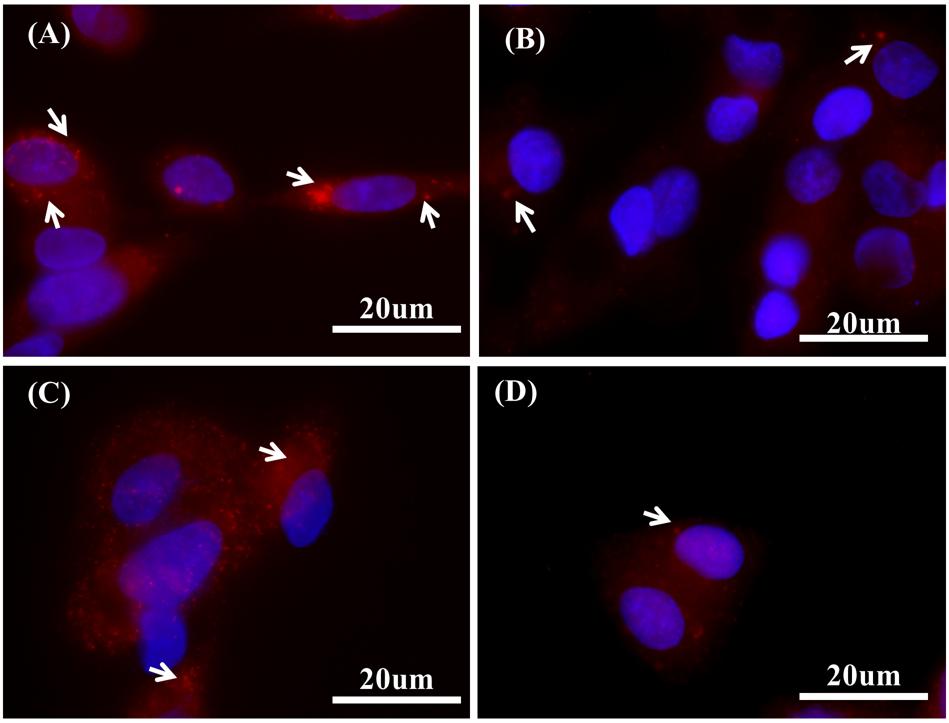

Supplement: Supplementary file 1 [file ijms-15-04299-s001.pdf]
